# Utilization of Supervised Machine Learning to Understand Kinase Inhibitor Toxophore Profiles

**DOI:** 10.3390/ijms24065088

**Published:** 2023-03-07

**Authors:** Andrew A. Bieberich, Christopher R. M. Asquith

**Affiliations:** 1 AsedaSciences Inc., 1281 Win Hentschel Boulevard, West Lafayette, IN 47906, USA; 2 School of Pharmacy, Faculty of Health Sciences, University of Eastern Finland, 70211 Kuopio, Finland; 3 Structural Genomics Consortium and Division of Chemical Biology and Medicinal Chemistry, UNC Eshelman School of Pharmacy, University of North Carolina, Chapel Hill, NC 27599, USA; 4 Department of Pharmacology, School of Medicine, University of North Carolina, Chapel Hill, NC 27599, USA

**Keywords:** kinase inhibitors, toxophore, machine learning drug discovery, 4-anilinoquinoline, 4-anilinoquinazoline

## Abstract

There have been more than 70 FDA-approved drugs to target the ATP binding site of kinases, mainly in the field of oncology. These compounds are usually developed to target specific kinases, but in practice, most of these drugs are multi-kinase inhibitors that leverage the conserved nature of the ATP pocket across multiple kinases to increase their clinical efficacy. To utilize kinase inhibitors in targeted therapy and outside of oncology, a narrower kinome profile and an understanding of the toxicity profile is imperative. This is essential when considering treating chronic diseases with kinase targets, including neurodegeneration and inflammation. This will require the exploration of inhibitor chemical space and an in-depth understanding of off-target interactions. We have developed an early pipeline toxicity screening platform that uses supervised machine learning (ML) to classify test compounds’ cell stress phenotypes relative to a training set of on-market and withdrawn drugs. Here, we apply it to better understand the toxophores of some literature kinase inhibitor scaffolds, looking specifically at a series of 4-anilinoquinoline and 4-anilinoquinazoline model libraries.

## 1. Introduction

Protein kinases catalyze the transfer of a phosphate group from adenosine triphosphate (ATP) to tyrosine, threonine, or serine residues in specific target substrates and proteins. These phosphorylation events are ubiquitous within signal transduction pathways and hence provide regulatory points for potential therapeutic intervention [1]. Kinases have been extensively investigated and successfully targeted for more than 30 years, with more than 70 kinase inhibitors clinically approved by the FDA [2,3]. While most of the currently approved drugs focus on multi-targeted tyrosine kinase inhibitors to treat cancer [3,4,5,6,7], the approval of kinase inhibitors to treat non-oncological related diseases, including rheumatoid arthritis, lung fibrosis, and psoriasis, has demonstrated a more extensive utility to treat human disease [8,9]. There are more than 500 kinases in the human genome [10], with only a small percentage targeted by currently approved drugs, highlighting a potential untapped opportunity in the remaining kinome [11]. Large-scale kinome-wide profiling of ATP-competitive kinase inhibitors has also started to uncover the preferred chemotypes for the inhibition of many of the relatively under-studied kinases or dark kinases [6,7,12,13,14,15]. Despite the success in the development of kinase inhibitor drugs, there is still a need for new inhibitors and heterocycles on which to build ATP-competitive inhibitors [11]. 

As available structural space is expanded to identify new inhibitors, the drug development pipeline would benefit from the added efficiency of conducting toxicity de-risking in parallel [16]. Screening candidate pharmaceuticals for the detection of potential toxicity mechanisms and safety risks is a field that has developed substantially during the past three decades [17]. Structure-based drug design matches small molecule structures to target binding sites [18,19]. Whereas polypharmacology-based toxicity screening detects interactions between small molecules and secondary biomolecular targets known to be associated with adverse drug reactions and hence, can be synergically beneficial [20,21,22]. Alternatively, cell-based multiparametric phenotypic screens can inform a similar de-risking process using relevant biological readouts [23]. Recently, these two strategies were directly compared for ranking the human safety risks within a set of 40 excipient compounds. The two methods produced complementary information, the phenotypic screen was less labor intensive and used a machine learning classifier to convert its multiparametric data into an easily interpretable risk score [24]. We subsequently employed this same phenotypic screen to rank estimated human safety risk for candidate kinase inhibitors targeting chordoma models [25]. We now describe the use of this screening method as a structure-activity relationship approach for assessing toxicity risk among a more generalized set of kinase inhibitors.

## 2. Results

We have previously published complete descriptions of the logical design and methodological execution for the AsedaSciences^®^ SYSTEMETRIC^®^ Cell Health Screen [24,25]. Briefly, it is a multiparametric live-cell phenotypic screen using automated flow cytometry (FC), in which a twelve-parameter acute cellular stress phenotype is classified by a supervised machine learning classifier. The classifier uses a multidimensional logistic regression model in which each dimension is an FC parameter. The training was performed with a 300-compound training set [24,25], which consisted of on-market and withdrawn drugs, research compounds, and several agricultural/industrial compounds [24,25]. The training set was first divided into binary outcome classes (high toxicity risk and low toxicity risk) using literature, clinical trial results, and market histories (where applicable). Next, all 300 compounds were processed through the FC screen and the empirical data populated distributions within each of the two known outcome classes. These distributions optimized the logistic regression model, defining the dependence of the outcome on each of the 12 FC parameters. The trained classifier subsequently classifies the acute cellular stress phenotype produced when an unknown test compound is applied to the cells. The final classification value, or Cell Health Index (CHI), is a probability value (0–1) representing the maximum likelihood that a test compound’s phenotype belongs in the high toxicity risk outcome class (Tables S1 and S2). In addition, the classifier can be used to produce the same type of probability score by using only one or a subset of the twelve FC parameters, and this is how it generates the biological fingerprint, comprised of eight phenotypic endpoints. Hence, for example, the cell morphology score is produced by allowing the classifier to only see four FC parameters related to forward scatter and side scatter from one laser. The assay is run with HL-60 cells, not because of any specific appropriateness as a disease model but for two pragmatic reasons. Suspension cell culture enables automated flow cytometry, and during the screen prototyping phase, HL-60 cells empirically produced an optimal dynamic range for the required fluorescent reporter dyes. This resulted in a screen design that was most generalizable across compounds from diverse therapeutic and chemical classes while having relatively low labor intensity and cost.

To have a better understanding of the kinase inhibitor toxophore landscape, we first screened thirty-one literature-reported, late-stage, and clinically approved kinase inhibitors in the Cell Health Screen (Table 1). The CHI results showed that twenty one inhibitors had higher risk factors, with four in the mid-range and six showing risk at or lower than 0.41. These results are, in part, reflective of on-target effects from these anti-cancer agents that generally target pathways promoting cell growth [26].

The six kinase inhibitors with lower toxicity risk are potentially the most interesting, as although many kinase inhibitors were developed towards specific targets, in practice, most of these drugs are multi-kinase inhibitors. That leveraging of the ATP pocket across multiple kinases increases their clinical efficacy, but it can also lead to increased toxicity. To target diseases outside of oncology, kinase inhibitors with lower toxicity and likely narrower target specificity are required [27]. This will enable the treatment of chronic diseases via kinase targets, including inflammation and neurodegeneration, potentially opening the route of personalized medicine [27,28,29].

These six kinase inhibitors with low toxicities include Tofacitinib and Ruxolitinib, which are both Janus kinase (JAK) inhibitors, based on the 7*H*-pyrrolo[2,3-*d*]pyrimidin-4-amine core scaffold [30,31]. Tofacitinib has been FDA-approved for a number of non-oncology indications, including the treatment of psoriatic arthritis, juvenile idiopathic arthritis, and ulcerative colitis [31,32,33]. Ruxolitinib has also been used to treat myelofibrosis, polycythemia vera, and steroid-refractory acute graft-versus-host disease [31,34]. Ruxolitinib has more recently been approved for several topical indications, including mild to moderate atopic dermatitis [35] and the treatment of vitiligo [36]. Tofacitinib and ruxolitinib are both primarily targeting non-oncology indications, which may, in part, explain these favorable CHI values, as their intended uses would not tolerate high human safety risk. Trametinib and dabrafenib were also in this group of six low-scoring kinase inhibitors. Trametinib is a highly selective allosteric mitogen-activated protein kinase kinase (MEK) inhibitor [37], that was originally approved for the treatment of malignant melanoma driven by the BRAF V600E mutation in combination with BRAF inhibitors, such as dabrafenib [38]. More recently, the combination of dabrafenib with trametinib has been approved for BRAF V600-positive advanced or metastatic non-small-cell lung cancer (NSCLC) [39]. These targeted therapies can be considered a first step towards personalized medicine, where the presence of the BRAF V600 mutation dictates the success of the treatment [40,41]. The narrower kinome spectrum of these compounds again may help to explain the favorable CHI values for both trametinib and dabrafenib.

The final two compounds of the six low-scoring kinase inhibitors were erlotinib, a first-generation epidermal growth factor receptor (EGFR) inhibitor [42], and sapitinib, a second-generation reversible EGFR inhibitor [43,44]. The main clinical indication for erlotinib is NSCLC, but there have also been subsequent approvals for the treatment of locally advanced, unresectable, or metastatic pancreatic cancer in combination with gemcitabine [45,46]. Whereas sapitinib has an enhanced pharmacologic profile due in part to equipotent inhibition of EGFR, erbB2, and erbB3, showing potent antitumor activity in preclinical cancer models [43,47]. Erlotinib and sapitinib are both oncology drugs based on the 4-anilinoquinazoline kinase inhibitor scaffold and are multi-kinase inhibitors, albeit not as promiscuous across the kinome as some inhibitors [6,7,12,13,14,15,16]. The fact that both of these compounds have a favorable CHI prompted us to further investigate the 4-anilinoquin(az)oline scaffold.

**Scheme 1:**
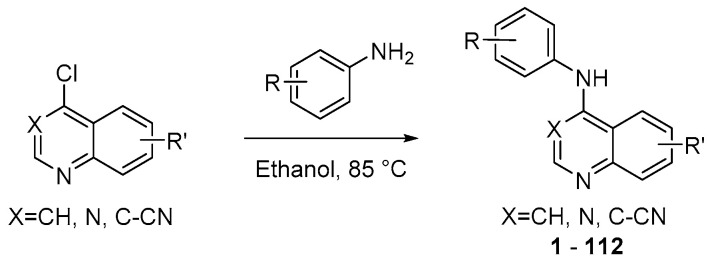
General synthetic procedure to access the 4-anilinoquin(az)olines.

To explore the 4-anilinoquin(az)oline scaffold, we profiled seven focused arrays of compounds, probing the toxicity profile structure-activity relationships of the quinoline/quinazoline scaffold. We synthesized and screened a series of compounds (**1**–**112**) to follow up on the results of erlotinib and sapatinib, exploring the 4-anilinoquin(az)oline through a series of nucleophilic aromatic displacements of 4-chloroquin(az)olines with a series of anilines in good yields (Scheme 1) consistent with previous reports [48,49,50,51,52,53].

To understand the structural drivers of toxicity on the 4-anilinoquin(az)oline scaffold, we first screened a series of simplified erlotinib-related 4-anilinoquinazolines containing the 3-ethynylaniline (**1**–**20**) (Table 2). We first screened *N*-(3-ethynylphenyl)-6,7-dimethoxyquinazolin-4-amine (**1**) and found that, despite the curtailment of the pendent arms with the removal of the ethylene glycol linker to afford the methoxy groups, the toxicity profile was broadly similar with a CHI of 0.41 (vs. 0.40 for erlotinib). Interestingly, the removal of the 7-position methoxy **2** or the 6-position methoxy **3** resulted in a decrease in the CHI, with **3** showing an almost 40% reduction in CHI. In the fingerprint of each compound, there appears to be a switch in driving CMI toxicity and limited reactive oxygen species (ROS) involvement in **2**, while in **3,** this trend is reversed. The catechol with the fused methyl spacer **4** cleaned the profile further, with a more than two-fold reduction of CHI compared with erlotinib. The extension of the fused spacer to ethyl **5** resulted in an almost 40% increase in the CHI compared with erlotinib, while the unsubstituted quinazoline **6** showed an increase of 80% in the CHI. The introduction of 6-position fluorine **7** reduced the CHI substantially compared to both the unsubstituted analog **6** and erlotinib. The 6,7-position difluoro **8** had a slightly lower CHI, at 0.26, compared to 0.31 for 6-fluoro **7**. The trend of 6-position halogens chloro **9**, bromo **10**, iodo **11,** and trifluoromethyl **12** analogs all showed a almost 50% reduction in CHI compared with erlotinib. Interestingly, switching the halogen from the 6- to 7-position led to an increase in CHI, with the 7-position fluoro **13** having the same CHI as erlotinib at 0.40. The 7-position chloro **14** and bromo **15** had similar fingerprints and an identical CHI of 0.33, an almost 20% reduction in CHI compared with erlotinib. However, unlike the 6-position analogs, the trend did not continue, with the 7-position iodo **16** and trifluoromethyl **17** having a nearly two-fold increase in CHI compared to erlotinib. Switching to the 7-position cyano **18** reduced CHI but still resulted in a 10% increase over erlotinib. The 6-position cyano analog **19** showed a more favorable CHI with more than 50% reduction relative to the 7-position cyano **18**. The 6-position methylsulfone **20** also performed favorably with a low CHI of 0.22.

Second, we screened a series of 3-cyanoquinolines containing the 3-ethynylaniline (**21**–**32**) (Table 3). The 3-cyanoquinoline still maintains the ability to form a dual hydrogen bond at the hinge region but forces the aniline out of plane to an almost perpendicular angle [51]. The unsubstituted analog **21** showed a 75% increase in CHI compared to erlotinib, similar to the quinazoline analog **6**. However, unlike the 6-position halogen quinazoline analogs **7** and **9**–**11**, the 3-cyanoquinoline derivatives **22**–**25** all showed similar or higher toxicity with relatively high CHI risk indicators. A switch to the 6-position methylsulfone **26** reduced the CHI by more than two-fold compared with erlotinib. A similar level of reduction was also seen in the CHI of the 6-position methoxy **27**, potentially related to the electron-donating ability of these two compounds. Screening 4-((3-ethynylphenyl)amino)-6,7-dimethoxyquinoline-3-carbonitrile (**28**), we found the same CHI as erlotinib despite several phenotypic endpoint score differences and differing structural features. The 7-position methoxy analog **29** showed a 10% increase in CHI, while the chloro substitution **30** showed a 10% decrease in CHI. The other two 7-position halogens had much higher CHI, with the bromo **31** 50% higher and the iodo **32** 100% higher compared with erlotinib. The 7-position iodo **32** had a similar profile to the quinazoline counterpart **16**.

Third, we switched the 3-ethynylaniline with the 3,4,5-trimethoxyaniline and screened a series of 3-cyanoquinolines (**33**–**43**) (Table 4), starting with 6,7-dimethoxy-4-((3,4,5-trimethoxyphenyl)amino)quinoline-3-carbonitrile (**33**) which had a similar CHI to erlotinib. The removal of either methoxy group **34**–**35** resulted in a 25% decrease in CHI with respect to **33** and erlotinib. The unsubstituted analog **36**, unlike the 3-ethynylaniline counterparts quinazoline **6** and 3-cyanoquinoline **21**, had a lower toxicity risk profile with a nearly two-fold reduction compared with erlotinib’s CHI. The same was the case with the 6-position halogens chloro **37**, bromo **38_,_** and iodo **39,** showing a 50–75% reduction in CHI compared with erlotinib. The 6-position methyl sulfone **40** showed a 50% spike in the CHI to 0.59, which was an opposite trend to the unsubstituted analogs, with counterparts quinazoline **20** and 3-cyanoquinoline **26** both having shown a lower toxicity risk estimate. Last, the 7-position halogen chloro **41**, bromo **42**, and iodo **43** all demonstrated a lower CHI than erlotinib. The chloro analog **41** showed a more than 50% reduction in CHI, with the bromo **42** and iodo **43** showing a more modest 10% reduction in CHI. This was, however, a much shallower trend than the matched pair quinazolines and 3-cyanoquinoline 3-ethynylaniline analogs, particularly with the iodo derivatives **16** and **32**.

Fourth, we switched from the 3-cyanoquinoline to a series of quinazolines while maintaining the 3,4,5-trimethoxyaniline (**44**–**62**) (Table 5). This time, the direct derivative **44** of erlotinib was screened with the 3,4,5-trimethoxyaniline replacement and found to have a 40% lower CHI compared to erlotinib. This effect appears to be mainly driven by a reduction in glutathione depletion (GSH) and a reduction in cell membrane disruption effects (CM). The 7-position mono-methoxy group derivative **45** showed a further reduction with a >50% lower CHI risk estimate than erlotinib, while the 6-position mono-methoxy **46** had only a 20% reduction in CHI. The unsubstituted analog **47** had the lowest CHI of the entire study at 0.13, which contrasted with some of the previous unsubstituted analogs, including **6** and **21**, but was more consistent with **36** that had the 3,4,5-trimethoxyaniline present in the compound. Interestingly, the addition of a methyl group in the 6-position **48** caused the CHI to double compared to the unsubstituted derivative **47**. Switching the methyl for a fluoro **49** maintained the CHI, as does having a 6,7-position difluoro substitution **50** and 6-position chloro **51**. However, increasing the size of the 6-position halogen appeared to be unfavored, with the bromo analog **52** having an almost 50% increase over the CHI of erlotinib and >100% increase in CHI from chloro **51** to bromo **52**. The penalty appears to plateau with the 6-position iodo **53** and trifluoromethyl **54** showing only a 10% uptick on the CHI of erlotinib. The 7-position halogens are more favored with low CHI across the fluoro **55**, chloro **56,** and bromo **57**. The 7-position iodo **58** reversed that trend with a 2.5-fold increase compared to bromo **57** and an almost 50% increase compared with erlotinib. The 7-position trifluoromethyl **59** was near parity with erlotinib, with only a 10% increase in CHI. Interestingly, switching to 7-position cyano **60** led to a more favorable CHI with a 60% reduction compared with erlotinib, while the 6-position cyano **61** showed parity with the CHI of erlotinib. Switching to the 6-position methyl sulfone **62** recovered the earlier gain from the 7-position cyano **60** with an identical CHI.

Fifth, building on the encouraging results of the 3,4,5-trimethoxyaniline analogs, we switched to another less common kinase hinge binder, quinoline (**63**–**80**) (Table 6). The quinoline has a reduced capacity to form an additional hydrogen bond in the 3-position of the ring system, but the C-H can push the aniline portion of the scaffold out of plane of the quinoline by up to 60 degrees [51]. Initially, the dimethoxy analog 6,7-dimethoxy-*N*-(3,4,5-trimethoxyphenyl)quinolin-4-amine (**63**) was screened and, despite a narrow spectrum on the kinome [48], afforded a CHI of close to with a 130% increase compared to erlotinib. The removal of the 6-position methoxy group to afford **64** reduced the CHI by almost 3-fold from the dimethoxy **63** to a much more favorable 0.32, a 20% reduction compared with erlotinib. Removal of the 7-position methoxy group to produce **65** reduced the CHI with respect to the dimethoxy **63**, but the CHI was still 40% greater than erlotinib.

The unsubstituted analog **66** was consistent with **36** and **47** and demonstrated a lower CHI of 0.32. The 6-position fluoro **67** and 6,7-position difluoro **68** analogs both have a similar CHI to the unsubstituted derivative **66** with roughly a 30% reduction compared to erlotinib. Increased size of the halogen resulted in an increased toxicity risk; the chloro derivative **69** had parity with erlotinib, while the bromo **70** showed a 15% increase in CHI and the iodo **71** showed nearly a 70% increase. The 6-position trifluoromethyl **72** returned the CHI to parity with erlotinib, while the introduction of a cyano **73** at the 6-position reduced the CHI by a further 40% to 0.22. While the direct methylsulfone analog **74** had a slightly shallower 30% reduction compared to trifluoromethyl **72**, this was still a 40% reduction over erlotinib. Switching to the 7-position with a fluoro substitution **75** was favorable, with a 50% reduction in CHI over erlotinib; conversely, the chloro analog **76** showed an almost 60% increase in CHI. The 7-position bromo **77** showed a >100% improvement over chloro **76** and 30% over erlotinib. The respective iodo **78** was closer to parity with erlotinib with only a 10% reduction; this reduction was extended with the direct trifluoromethyl replacement to afford **79** with a 25% reduction. The 7-position cyano analog **80** showed an additional improvement with a 45% reduction in CHI compared with erlotinib, with the majority of the CHI appearing to be derived from nuclear membrane integrity 1 (NMI1).

Sixth, after we observed different profiles between the 3-ethynylaniline and 3,4,5-trimethoxyaniline, we selected the 6-(trifluoromethyl)quinoline, whose CHI was not only similar to erlotinib but has been shown to maintain cellular penetrance using a nanoBRET in-cell target engagement assay [48,49,50,51,52,53]. We fixed the quinoline and assessed how a series of point changes on the pendent aniline altered the toxicity profile of the scaffold (**81**–**100**) (Table 7). A direct replacement of the methoxy groups in **72** with fluorine to afford a 3,4,5-trifluoroaniline **81**, resulting in a compound that had an almost 40% lower CHI than erlotinib. The 4-position mono-fluoro **82** was nearly 30% lower, while the 3-position fluoro **83** jumped to 50% lower with a CHI of 0.21 compared to the CHI of 0.40 for erlotinib. The 2-position mono-fluoro **84** changed the trend and showed a 4-fold increase in CHI compared with the 3-position analog **83** and an almost 100% increase compared with the CHI of erlotinib. The 4-chloro-3-fluoroaniline derivative **85** had a similar CHI to the 2-position mono-fluoro **84** and with gefitinib (CHI = 0.80). Moving the chlorine around the ring to afford the 3-chloro-5-fluoroaniline derivative **86** reduced the CHI to 0.51, still about 25% higher than erlotinib.

The *N*-(3,4-dichlorophenyl)-6-(trifluoromethyl)quinolin-4-amine (**87**) analog showed a significant increase in CHI with over an 80% increase compared with the erlotinib. The 4-position mono-chloro **88** also had a substantial increase of more than 110% relative to the CHI of erlotinib, while the 3-position chloro **89** showed an almost 3-fold drop in CHI compared to the 4-position analog **88**. The 3-position analog had a decrease of >30% in CHI compared with erlotinib. Moving the chlorine around the ring to the 2-position **90** afforded a compound with a similar profile to the 4-position derivative **88**, where the CHI was 100% increased relative to the CHI for erlotinib. The 4-, 3- and 2-position bromo substitutions, **91**–**93,** respectively, showed consistent results with the choro analogs **88**–**90**. However, the larger 3-position iodo **94** broke the trend with over a 50% increase in CHI compared with erlotinib. Interestingly, the introduction of a cyano group at the 4-position **95** was well tolerated with a near 30% reduction in CHI compared with erlotinib, while the 3-position derivative **96** demonstrated a >40% decrease, with the 2-position analog **97** showing just over 20%. The cyano groups followed the same trend as the halogens, albeit with a less pronounced gradient. The 3-position trifluoromethyl **98** was consistent with the iodo **94**, likely related to size and/or electronegativity increase [54,55] and showed a further increased toxicity risk with a CHI of 0.81, >100% of the corresponding CHI of erlotinib. The 3-ethynylaniline derivative **99** was closer to the 3-cyanoquinoline series than the quinazoline, with a 75% increase in CHI in the presence of a 6-position halogen situated on the quinoline. This difference could be related to both electronics and the overall conformation of the scaffold [51,54]. The final analog in this series was the 4-((methylsulfonyl)methyl)aniline derivative **100**, which showed a substantially lower CHI with a drop of >40% compared with erlotinib.

Finally, we investigated the direct contribution of the methoxy groups on the aniline ring system of **72**, with a series of matched pair analogs (**101**–**112**) (Table 8). The removal of the central 4-position methoxy to afford **101** resulted in a compound that had a 60% increase in CHI compared with parent **72**. Removing one of the flanking methoxy groups (3-position) to afford **102** had a less pronounced effect on the CHI with a marginal 10% increase compared with parent **72**. Having the two methoxy groups in the 2,4-position, **103** was even more favorable, with a net reduction in toxicity risk of 30% compared with parent **72**. Intriguingly moving one of the methoxy groups to establish a 2,5-position **104** orientation actually caused a 3-fold increase in toxicity compared with the 2,4-position analog **104** and >100% compared with the CHI of parent **72**. The 4-position mono-methoxy **105** showed a favorable toxicity profile with a 20% reduction of the CHI compared with parent **72**. The 3-position analog had parity with parent **72**, while the 2-position showed a large increase of 100% in CHI compared with parent **72**, consistent with the other derivative containing a 2-position methoxy substitution **104**. Fusing the 3,4-dimethoxy analog with a methyl spacer to afford **106** was disfavored with an almost >70% premium compared with both parent **72** and **102**. The use of an ethyl bridging group provided for a more favorable CHI, where the increase was reduced to only 20%.

The direct switch to a quinazoline **110** from quinoline **72** led to a small 10% increase in CHI, but the removal of the central 4-position methoxy was more favorable on the quinazoline **111** than quinoline **101** and showed an almost 30% reduction compared with parent **72** and almost 90% compared with **101**. The final analog in this series **112**, the 3-position methoxy, showed a 50% drop in CHI compared to quinoline **106** and parent **72**.

## 3. Discussion

There are many barriers to developing a successful lead compound and eventual clinical candidate [19,56,57]. Here we presented a toxicity profiling platform to accelerate the drug discovery process. This screening allows granular detail and insights into the toxicity profile within a scaffold series [24,25]. We previously showcased a series of kinase inhibitor-based optimization projects within these 4-anilinoquin(az)olines series [48,49,50,51,52,53]. We now demonstrate detailed output from thirty-one clinically used kinase inhibitors along with seven discrete series of 4-anilinoquin(az)olines totaling over 100 cell active compounds [48,49,50,51,52,53]. New chemical approaches and molecular insight into the development of highly selective and non-toxic kinase inhibitors are required in order to facilitate targeting non-oncology-based indications within the kinome [26]. This screening could enable a better understanding of how unknown toxicity liabilities can be identified earlier in the drug discovery process. The early understanding of potential latent toxophores could not only have potential implications across different kinase scaffolds but also more widely within medicinal chemistry.

The in-depth screening around erlotinib and sapatinib focused on several different characteristics, primarily the cone angle of the aniline vs. the quin(az)oline, where the aniline can be in or out of plane, and the electronics/sterics of both the aniline and quin(az)oline substitution [51,54]. A number of interesting trends were observed, including the favorability of the 3-position on the aniline ring system, which disproportionately afforded fewer toxic results. These included a trend of 3-position halogens where fluoro **83**, chloro **89**, bromo **93,** and even 3-chloro-5-fluoro **86** formed non-toxic local minima compared to their respective 2- and 4- position counterparts. There are also some results with real-world applications, such as the case of 4-chloro-3-fluoro **85**, the reversed gefitinib aniline derivative that has a similar CHI to gefitinib (CHI = 0.80). Other matched pairs provide further useful structure/toxicity lessons, e.g., erlotinib and the 6,7-dimethoxy analog **1** indicate that these extended 6,7-positions pendant arms have limited influence on core scaffold toxicity. This observation would also support a kinase binding hypothesis, as the extensions on the 6,7-positions are solvent exposed. This solvent-exposed observation is supported by a number of co-crystal structures of 4-anilinoquin(az)oline, including bosutinib in Src, pelitinib in PKMYT1, and erlotinib in EGFR, among others [58,59,60]. Interestingly, the first two atoms in the point of attachment of the solvent-exposed 6,7-position region did heavily influence the series toxicity profiles; this could be due to these atoms dictating the electronic contribution of the substituent to the quin(az)oline ring system.

This work also expands the knowledge base around the biologically relevant 4-anilinoquin(az)oline scaffold more generally, with extensive examples of medicinally relevant quinolines and quinazolines reported in the literature. These include CB2 receptor agonists [61], anti-tuberculosis compounds [62], anti-malarial compounds, such as amodiaquine [63], and compounds active against the protozoan parasite *Trypanosoma brucei* [64]. There has also been increased investigation around the anti-viral potential of the 4-anilinoquin(az)oline scaffold and a series of viruses, including HMCV [65], DENV [66], VEEV [67], and more recently, SARS-CoV-2 infection [68].

## 4. Materials and Methods

### 4.1. Biology

The AsedaSciences^®^ SYSTEMETRIC^®^ Cell Health Screen was performed as previously described (see Supplementary Information) [24,25].

### 4.2. Chemistry

All reactions were performed using flame-dried round-bottomed flasks or reaction vessels unless otherwise stated. Where appropriate, reactions were carried out under a nitrogen atmosphere with dry solvents unless otherwise stated. Yields refer to chromatographically and spectroscopically pure isolated yields. Reagents were purchased at the highest commercial quality and used without further purification unless otherwise stated. Reactions were monitored by thin-layer chromatography carried out on 0.25 mM E. Merck silica gel plates (60_F-254_) using ultraviolet light as visualizing agent. NMR spectra were recorded on a Varian Inova 400 or Inova 500 spectrometer and were calibrated using residual protic solvent as an internal reference. The following abbreviations or combinations thereof were used to explain the multiplicities observed: s = singlet, d = doublet, t = triplet, q = quartet, m = multiplet, and br = broad. Liquid chromatography (LC) and high-resolution mass spectra (HRMS) were recorded on a ThermoFisher hybrid LTQ FT (ICR 7T). The LC-HRMS was collected as previously described [51].

#### General Procedure for the Synthesis of 4-Anilinoquin(az)olines

4-chloroquin(az)oline derivative (1.0 eq.), and aniline derivative (1.1 eq.), were suspended in ethanol (10 mL) and refluxed for 18 h. The crude mixture was purified by flash chromatography using EtOAc:hexane followed by 1–5 % methanol in EtOAc. The solvent was removable under reduced pressure; the product was obtained as a free following solid or recrystallized from ethanol/water. Compounds were synthesized as previously described: **1**–**3** [53], **4**–**26** [51], **27** [53], **28** [52], **29** [53], **30**–**32** [51], **33** [52], **34**–**43** [51], **44** [52], **45**–**62** [51], **63**–**67** [48], **68 [51]**, **69**–**71** [49], **72**–**75** [48], **76**–**78** [50], **79**–**112** [48].

## 5. Conclusions

The kinase inhibitor field is rapidly expanding along with the potential therapeutic benefit, but to create successful clinical candidates, a clear understanding of the latent toxicity profile is imperative. This is particularly acute in the case of treating other kinase indications beyond oncology, such as chronic diseases with kinase targets, including neurodegeneration and inflammation. The screening platform employed for this study enables a better understanding of the latent toxophores potentially within some literature kinase inhibitor scaffolds, enabling the more effective design of selective non-toxic inhibitors. In addition to this, we have provided a series of comprehensive data sets on the 4-anilinoquin(az)oline scaffold to enable more effective design, a better understanding of this chemotype, and expansion of the medicinal chemist’s toolbox.

## Figures and Tables

**Table 1:** Toxicity profiling of a broad range of clinical and literature kinase inhibitors ^a,b,c^.

Name	CM	CMI	ROS	GSH	NMI1	CC	NMI2	MMP	CHI
**Ponatinib**	1.00	1.00	1.00	1.00	1.00	0.95	0.99	1.00	0.95
**Dacomitinib**	1.00	1.00	1.00	1.00	1.00	0.98	0.99	1.00	0.94
**Bosutinib**	1.00	1.00	1.00	1.00	1.00	0.91	1.00	1.00	0.94
**Vandetanib**	1.00	1.00	1.00	1.00	1.00	0.94	1.00	1.00	0.93
**Osimertinib**	1.00	1.00	1.00	1.00	0.99	0.96	0.99	1.00	0.93
**Palbociclib**	1.00	1.00	1.00	1.00	1.00	0.98	0.99	1.00	0.93
**Sunitinib**	1.00	1.00	1.00	1.00	1.00	0.93	0.99	1.00	0.93
**Crizotinib**	1.00	1.00	1.00	1.00	1.00	0.96	0.99	1.00	0.92
**Canertinib**	1.00	1.00	1.00	1.00	1.00	0.68	1.00	1.00	0.92
**Afatinib**	1.00	1.00	1.00	1.00	1.00	0.93	0.99	1.00	0.92
**Tesevatinib**	1.00	1.00	1.00	1.00	0.91	0.96	0.99	1.00	0.92
**Cobimetinib**	1.00	1.00	1.00	1.00	1.00	0.95	1.00	1.00	0.91
**Pelitinib**	1.00	1.00	1.00	1.00	0.77	0.95	0.99	1.00	0.91
**Ibrutinib**	1.00	1.00	1.00	1.00	0.97	0.99	0.98	1.00	0.90
**Lapatinib**	1.00	1.00	0.90	0.96	0.97	0.93	0.92	1.00	0.89
**Sorafenib**	1.00	1.00	1.00	0.99	1.00	0.87	0.99	1.00	0.88
**Neratinib**	1.00	1.00	1.00	0.98	0.97	0.83	1.00	1.00	0.87
**Regorafenib**	0.99	0.97	1.00	0.89	1.00	0.89	0.98	1.00	0.85
**Gefitinib**	1.00	0.99	0.93	0.97	0.61	0.93	0.94	0.99	0.80
**Poziotinib**	1.00	0.99	0.40	0.98	0.98	0.97	0.89	0.99	0.80
**Saracatinib**	1.00	0.92	0.72	0.93	0.81	0.76	0.78	0.71	0.69
**Alectinib**	1.00	0.98	0.99	0.97	0.84	0.054	0.036	0.15	0.55
**Vemurafenib**	0.77	0.52	0.95	0.22	0.99	0.31	0.89	0.37	0.51
**Cabozantinib**	0.94	0.73	0.080	0.029	0.49	0.17	0.77	0.70	0.48
**Pazopanib**	0.73	0.12	0.73	0.22	0.27	0.34	0.078	0.71	0.46
**Trametinib**	0.86	0.83	0.34	0.15	0.53	0.21	0.073	0.10	0.41
**Erlotinib**	0.77	0.50	0.13	0.63	0.10	0.10	0.062	0.12	0.40
**Ruxolitinib**	0.90	0.19	0.45	0.089	0.16	0.30	0.061	0.23	0.39
**Sapitinib**	0.63	0.30	0.024	0.28	0.32	0.43	0.28	0.22	0.38
**Dabrafenib**	0.37	0.50	0.22	0.089	0.21	0.49	0.058	0.45	0.37
**Tofacitinib**	0.36	0.042	0.034	0.032	0.056	0.11	0.14	0.12	0.23

^a^ Abbreviations from columns left to right. CM: Cell morphology; CMI: Cell membrane integrity; ROS: Reactive oxygen species; GSH: Glutathione; NMI1: Nuclear membrane integrity 1; CC: Cell Cycle; NMI2: Nuclear membrane integrity 2; MMP: Mitochondrial membrane potential; CHI: Cell Health Index. ^b^ Traffic light colouring matching classifier values (Red–Green: high toxicity risk–low toxicity risk). ^c^ all *n* = 2.

**Table 2:** Toxicity profiling of erlotinib and related simplified quinazolines (**1**–**20**) ^a,b,c^.

	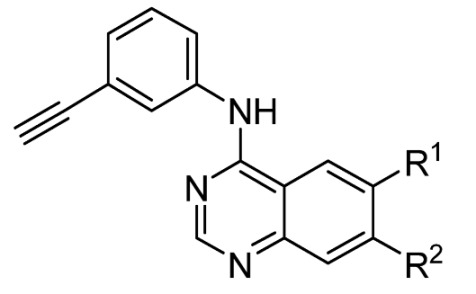										
Name	R^1^	R^2^	CM	CMI	ROS	GSH	NMI1	CC	NMI2	MMP	CHI
**Erlotinib**	6,7-(OCH_2_CH_2_OMe)_2_		0.77	0.50	0.13	0.63	0.10	0.10	0.062	0.12	0.40
**1**	OMe	OMe	0.85	0.11	0.32	0.18	0.47	0.086	0.41	0.39	0.41
**2**	OMe	H	0.24	0.62	0.17	0.038	0.42	0.38	0.068	0.019	0.31
**3**	H	OMe	0.050	0.032	0.63	0.0085	0.61	0.52	0.15	0.068	0.25
**4**	OCH_2_O		0.0019	0.0038	0.027	0.087	0.72	0.13	0.0045	0.016	0.18
**5**	OCH_2_CH_2_O		0.053	0.91	0.85	0.39	0.73	0.77	0.83	0.91	0.56
**6**	H	H	0.96	0.99	0.98	0.98	0.53	0.86	0.76	1.00	0.74
**7**	F	H	0.0051	0.21	0.20	0.12	0.059	0.65	0.17	0.49	0.31
**8**	F	F	0.0041	0.091	0.34	0.087	0.082	0.34	0.11	0.26	0.26
**9**	Cl	H	0.0084	0.0042	0.073	0.036	0.037	0.19	0.028	0.029	0.16
**10**	Br	H	0.0047	0.030	0.20	0.011	0.13	0.10	0.053	0.015	0.16
**11**	I	H	0.0037	0.0024	0.083	0.050	0.13	0.087	0.020	0.11	0.19
**12**	CF_3_	H	0.0063	0.38	0.088	0.029	0.069	0.071	0.054	0.013	0.21
**13**	H	F	0.058	0.045	0.77	0.0040	0.21	0.59	0.19	0.74	0.40
**14**	H	Cl	0.045	0.079	0.52	0.010	0.20	0.13	0.018	0.25	0.33
**15**	H	Br	0.024	0.41	0.86	0.010	0.033	0.74	0.30	0.17	0.33
**16**	H	I	1.00	1.00	1.00	0.98	0.21	0.95	0.94	0.99	0.77
**17**	H	CF_3_	1.00	0.87	1.00	0.79	0.60	0.92	0.93	1.00	0.78
**18**	H	CN	0.43	0.052	0.41	0.040	0.37	0.25	0.10	0.10	0.45
**19**	CN	H	0.0026	0.0079	0.042	0.023	0.33	0.27	0.094	0.11	0.18
**20**	SO_2_Me	H	0.0013	0.0057	0.055	0.044	0.36	0.33	0.089	0.21	0.22

^a^ Abbreviations from columns left to right. CM: Cell morphology; CMI: Cell membrane integrity; ROS: Reactive oxygen species; GSH: Glutathione; NMI1: Nuclear membrane integrity 1; CC: Cell Cycle; NMI2: Nuclear membrane integrity 2; MMP: Mitochondrial membrane potential; CHI: Cell Health Index. ^b^ Traffic light colouring matching classifier values (Red–Green: high toxicity risk–low toxicity risk). ^c^ all *n* = 2.

**Table 3:** Toxicity profiling of 3-ethynylaniline analogs containing a 3-cyanoquinoline scaffold, similar to Bosutinib (**21**–**32**) ^a,b,c^.

	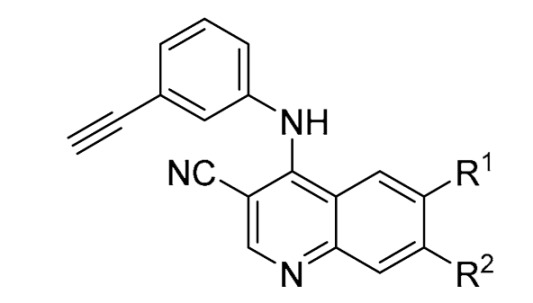										
Name	R^1^	R^2^	CM	CMI	ROS	GSH	NMI1	CC	NMI2	MMP	CHI
**Bosutinib**	-		1.00	1.00	1.00	1.00	1.00	0.91	1.00	1.00	0.94
**Erlotinib**	-		0.77	0.50	0.13	0.63	0.10	0.10	0.062	0.12	0.40
**21**	H	H	0.99	1.00	0.98	0.98	0.85	0.76	0.81	0.97	0.70
**22**	F	H	0.98	0.99	0.93	0.97	0.71	0.76	0.72	0.96	0.69
**23**	Cl	H	1.00	1.00	1.00	0.99	0.98	0.93	0.97	0.99	0.83
**24**	Br	H	1.00	1.00	1.00	0.98	0.32	0.98	0.98	0.96	0.75
**25**	I	H	0.88	1.00	0.89	0.93	0.74	0.91	0.81	0.99	0.70
**26**	SO_2_Me	H	0.0027	0.037	0.046	0.039	0.18	0.071	0.0093	0.021	0.16
**27**	OMe	H	0.020	0.004	0.056	0.0085	0.30	0.74	0.11	0.28	0.26
**28**	OMe	OMe	0.35	0.77	0.81	0.071	0.67	0.33	0.29	0.39	0.40
**29**	H	OMe	0.97	0.90	0.68	0.78	0.20	0.81	0.16	0.22	0.44
**30**	H	Cl	0.31	0.25	0.44	0.065	0.69	0.17	0.041	0.080	0.35
**31**	H	Br	0.95	0.88	0.87	0.92	0.45	0.92	0.34	0.84	0.59
**32**	H	I	1.00	1.00	0.99	0.98	0.95	0.95	0.94	0.99	0.81

^a^ Abbreviations from columns left to right. CM: Cell morphology; CMI: Cell membrane integrity; ROS: Reactive oxygen species; GSH: Glutathione; NMI1: Nuclear membrane integrity 1; CC: Cell Cycle; NMI2: Nuclear membrane integrity 2; MMP: Mitochondrial membrane potential; CHI: Cell Health Index. ^b^ Traffic light colouring matching classifier values (Red–Green: high toxicity risk–low toxicity risk). ^c^ all *n* = 2.

**Table 4:** Toxicity profiling of 3,4,5-trimethoxyaniline analogs containing a 3-cyanoquinoline scaffold similar to Bosutinib (**33**–**43**) ^a,b,c^.

	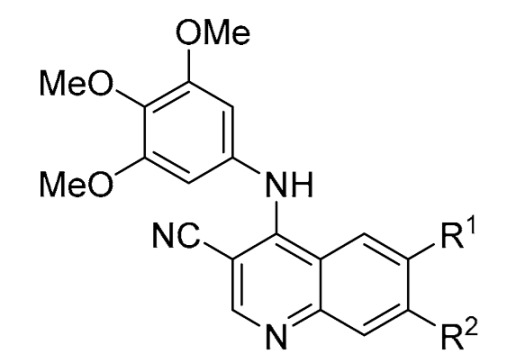										
Name	R^1^	R^2^	CM	CMI	ROS	GSH	NMI1	CC	NMI2	MMP	CHI
**Bosutinib**	-		1.00	1.00	1.00	1.00	1.00	0.91	1.00	1.00	0.94
**Erlotinib**	-		0.77	0.50	0.13	0.63	0.10	0.10	0.062	0.12	0.40
**33**	OMe	OMe	0.25	0.041	0.062	0.070	0.37	0.15	0.21	0.50	0.42
**34**	H	OMe	0.71	0.029	0.050	0.018	0.30	0.091	0.16	0.31	0.33
**35**	OMe	H	0.024	0.46	0.075	0.10	0.41	0.34	0.017	0.041	0.29
**36**	H	H	0.0021	0.57	0.072	0.042	0.13	0.35	0.047	0.019	0.23
**37**	Cl	H	0.19	0.013	0.28	0.010	0.20	0.35	0.073	0.093	0.25
**38**	Br	H	0.0063	0.12	0.36	0.019	0.067	0.29	0.078	0.065	0.29
**39**	I	H	0.033	0.044	0.084	0.025	0.16	0.12	0.15	0.16	0.27
**40**	SO_2_Me	H	0.52	0.82	0.10	0.30	0.90	0.95	0.78	0.96	0.59
**41**	H	Cl	0.25	0.021	0.22	0.012	0.55	0.060	0.026	0.15	0.27
**42**	H	Br	0.33	0.037	0.56	0.013	0.67	0.15	0.0063	0.18	0.36
**43**	H	I	0.013	0.23	0.37	0.14	0.58	0.13	0.33	0.21	0.37

^a^ Abbreviations from columns left to right. CM: Cell morphology; CMI: Cell membrane integrity; ROS: Reactive oxygen species; GSH: Glutathione; NMI1: Nuclear membrane integrity 1; CC: Cell Cycle; NMI2: Nuclear membrane integrity 2; MMP: Mitochondrial membrane potential; CHI: Cell Health Index. ^b^ Traffic light colouring matching classifier values (Red–Green: high toxicity risk–low toxicity risk). ^c^ all *n* = 2.

**Table 5:** Toxicity profiling of 3,4,5-trimethoxyaniline analogs containing a quinazoline scaffold, similar to Erlotinib and Sapitinib (**44**–**62**) ^a,b,c^.

	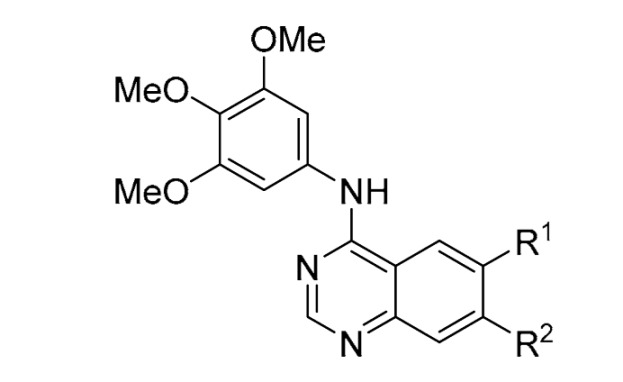										
Name	R^1^	R^2^	CM	CMI	ROS	GSH	NMI1	CC	NMI2	MMP	CHI
**Erlotinib**	-		0.77	0.50	0.13	0.63	0.10	0.10	0.062	0.12	0.40
**44**	6,7-(OCH_2_CH_2_OMe)_2_		0.37	0.38	0.0051	0.027	0.031	0.24	0.040	0.023	0.24
**45**	H	OMe	0.038	0.016	0.040	0.035	0.14	0.24	0.025	0.013	0.18
**46**	OMe	H	0.55	0.22	0.21	0.10	0.63	0.40	0.088	0.013	0.32
**47**	H	H	0.0078	0.0021	0.075	0.0074	0.25	0.059	0.034	0.052	0.13
**48**	Me	H	0.11	0.40	0.084	0.15	0.10	0.46	0.31	0.34	0.32
**49**	F	H	0.036	0.55	0.28	0.49	0.11	0.46	0.089	0.066	0.35
**50**	F	F	0.029	0.53	0.091	0.42	0.081	0.071	0.026	0.033	0.27
**51**	Cl	H	0.011	0.71	0.10	0.34	0.49	0.18	0.23	0.047	0.27
**52**	Br	H	0.99	0.88	0.085	0.84	0.90	0.80	0.60	0.52	0.57
**53**	I	H	0.88	0.77	0.074	0.53	0.47	0.82	0.52	0.26	0.45
**54**	CF_3_	H	0.81	0.43	0.065	0.38	0.75	0.80	0.47	0.47	0.45
**55**	H	F	0.029	0.0049	0.031	0.029	0.43	0.073	0.17	0.028	0.15
**56**	H	Cl	0.0049	0.37	0.45	0.14	0.68	0.33	0.14	0.12	0.28
**57**	H	Br	0.00066	0.14	0.28	0.014	0.30	0.10	0.23	0.11	0.22
**58**	H	I	0.97	0.97	0.74	0.93	0.18	0.80	0.80	0.64	0.57
**59**	H	CF_3_	0.087	0.74	0.85	0.66	0.18	0.68	0.80	0.53	0.44
**60**	H	CN	0.0065	0.21	0.13	0.035	0.13	0.015	0.039	0.0067	0.16
**61**	CN	H	0.71	0.51	0.27	0.56	0.77	0.066	0.073	0.031	0.38
**62**	SO_2_Me	H	0.0026	0.16	0.24	0.0062	0.30	0.022	0.060	0.0028	0.16

^a^ Abbreviations from columns left to right. CM: Cell morphology; CMI: Cell membrane integrity; ROS: Reactive oxygen species; GSH: Glutathione; NMI1: Nuclear membrane integrity 1; CC: Cell Cycle; NMI2: Nuclear membrane integrity 2; MMP: Mitochondrial membrane potential; CHI: Cell Health Index. ^b^ Traffic light colouring matching classifier values (Red–Green: high toxicity risk–low toxicity risk). ^c^ all *n* = 2.

**Table 6:** Toxicity profiling of 3,4,5-trimethoxyaniline analogs containing a quinoline scaffold (**63**–**80**) ^a,b,c^.

	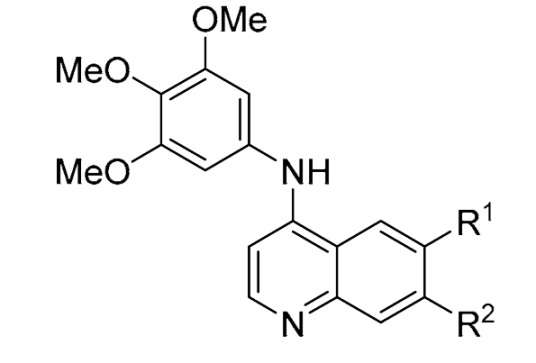										
Name	R^1^	R^2^	CM	CMI	ROS	GSH	NMI1	CC	NMI2	MMP	CHI
**Erlotinib**	-		0.77	0.50	0.13	0.63	0.10	0.10	0.062	0.12	0.40
**63**	OMe	OMe	1.00	1.00	1.00	1.00	0.99	0.99	0.98	1.00	0.92
**64**	H	OMe	0.85	0.038	0.56	0.35	0.33	0.070	0.024	0.089	0.32
**65**	OMe	H	0.91	0.87	0.15	0.87	0.92	0.95	0.57	0.49	0.56
**66**	H	H	0.18	0.32	0.022	0.29	0.20	0.68	0.0053	0.022	0.32
**67**	F	H	0.011	0.13	0.052	0.28	0.094	0.26	0.051	0.034	0.27
**68**	F	F	0.036	0.048	0.25	0.24	0.12	0.16	0.025	0.088	0.29
**69**	Cl	H	0.59	0.89	0.098	0.60	0.46	0.37	0.13	0.059	0.38
**70**	Br	H	0.87	0.69	0.25	0.26	0.62	0.71	0.77	0.33	0.46
**71**	I	H	0.97	0.97	0.94	0.92	0.76	0.98	0.89	0.91	0.67
**72**	CF_3_	H	0.89	0.78	0.063	0.096	0.24	0.50	0.064	0.078	0.37
**73**	CN	H	0.079	0.006	0.055	0.047	0.23	0.18	0.066	0.045	0.22
**74**	SO_2_Me	H	0.096	0.072	0.23	0.10	0.53	0.36	0.051	0.14	0.25
**75**	H	F	0.010	0.15	0.31	0.020	0.13	0.045	0.005	0.060	0.21
**76**	H	Cl	0.81	0.98	0.95	0.97	0.71	0.88	0.566	0.94	0.63
**77**	H	Br	0.28	0.12	0.11	0.15	0.28	0.052	0.045	0.021	0.27
**78**	H	I	0.097	0.11	0.18	0.18	0.22	0.51	0.024	0.15	0.35
**79**	H	CF_3_	0.29	0.078	0.27	0.21	0.05	0.18	0.0050	0.33	0.30
**80**	H	CN	0.010	0.035	0.12	0.027	0.76	0.026	0.0092	0.018	0.22

^a^ Abbreviations from columns left to right. CM: Cell morphology; CMI: Cell membrane integrity; ROS: Reactive oxygen species; GSH: Glutathione; NMI1: Nuclear membrane integrity 1; CC: Cell Cycle; NMI2: Nuclear membrane integrity 2; MMP: Mitochondrial membrane potential; CHI: Cell Health Index. ^b^ Traffic light colouring matching classifier values (Red–Green: high toxicity risk–low toxicity risk). ^c^ all *n* = 2.

**Table 7:** Toxicity profiling of different aniline substitutions of 4-anilinoquinolines (**81**–**100**) based on the scaffold of analog **72** ^a,b,c^.

	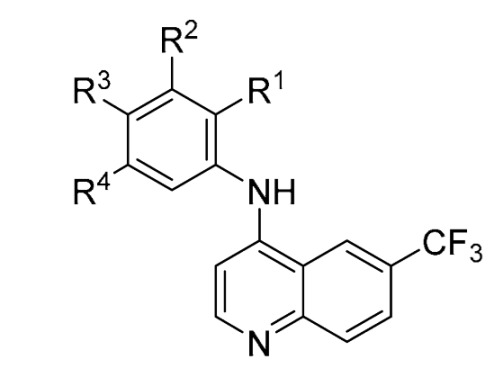												
Name	R^1^	R^2^	R^3^	R^4^	CM	CMI	ROS	GSH	NMI1	CC	NMI2	MMP	CHI
**Erlotinib**	-				0.77	0.50	0.13	0.63	0.10	0.10	0.062	0.12	0.40
**72**	H	OMe	OMe	OMe	0.89	0.78	0.063	0.096	0.24	0.50	0.064	0.078	0.37
**81**	H	F	F	F	0.059	0.16	0.84	0.036	0.31	0.053	0.087	0.056	0.25
**82**	H	H	F	H	0.096	0.058	0.85	0.062	0.69	0.082	0.063	0.15	0.29
**83**	H	F	H	H	0.00033	0.60	0.19	0.21	0.78	0.37	0.003	0.004	0.21
**84**	F	H	H	H	1.00	1.00	0.98	0.99	0.61	0.98	0.86	0.99	0.78
**85**	H	F	Cl	H	1.00	1.00	1.00	0.99	0.98	0.98	0.94	0.97	0.80
**86**	H	F	H	Cl	0.79	0.94	0.47	0.88	0.87	0.74	0.16	0.64	0.51
**87**	H	Cl	Cl	H	0.98	1.00	1.00	0.99	0.99	0.96	0.84	0.98	0.73
**88**	H	H	Cl	H	1.00	1.00	1.00	1.00	0.86	0.99	0.94	0.99	0.85
**89**	H	Cl	H	H	0.0070	0.89	0.066	0.46	0.85	0.084	0.0051	0.028	0.27
**90**	Cl	H	H	H	1.00	1.00	1.00	0.99	0.92	0.99	0.93	0.94	0.81
**91**	H	H	Br	H	0.99	1.00	1.00	1.00	0.98	0.96	0.91	0.99	0.75
**92**	H	Br	H	H	0.47	0.98	0.93	0.94	0.80	0.19	0.068	0.082	0.36
**93**	Br	H	H	H	1.00	1.00	1.00	1.00	0.60	0.97	0.95	0.96	0.75
**94**	H	I	H	H	0.95	0.99	0.97	0.97	0.90	0.89	0.26	0.83	0.62
**95**	H	H	CN	H	0.047	0.92	0.10	0.15	0.64	0.077	0.0064	0.072	0.29
**96**	H	CN	H	H	0.0066	0.50	0.37	0.34	0.65	0.056	0.0051	0.042	0.23
**97**	CN	H	H	H	0.27	0.68	0.63	0.23	0.55	0.47	0.017	0.051	0.33
**98**	H	CF_3_	H	H	1.00	1.00	1.00	0.99	0.95	0.99	0.91	0.98	0.81
**99**	H	C≡C	H	H	0.97	0.97	0.82	0.95	0.47	0.89	0.92	0.99	0.70
**100**	H	H	CH_2_SO_2_CH_3_	H	0.019	0.32	0.024	0.027	0.34	0.077	0.048	0.014	0.24

^a^ Abbreviations from columns left to right. CM: Cell morphology; CMI: Cell membrane integrity; ROS: Reactive oxygen species; GSH: Glutathione; NMI1: Nuclear membrane integrity 1; CC: Cell Cycle; NMI2: Nuclear membrane integrity 2; MMP: Mitochondrial membrane potential; CHI: Cell Health Index. ^b^ Traffic light colouring matching classifier values (Red–Green: high toxicity risk–low toxicity risk). ^c^ all *n* = 2.

**Table 8:** Toxicity profiling of different oxygen substituted anilines based on the scaffold of analog **72** (**101**–**112**) ^a,b,c^.

		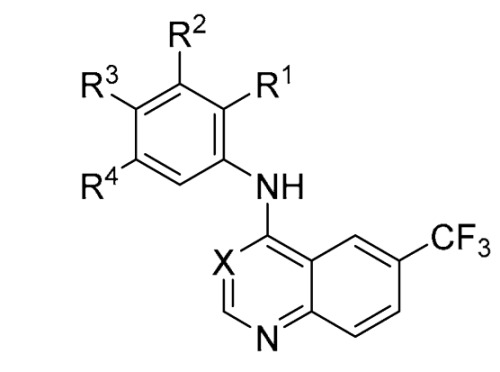												
Name	X	R^1^	R^2^	R^3^	R^4^	CM	CMI	ROS	GSH	NMI1	CC	NMI2	MMP	CHI
**Erlotinib**	-					0.77	0.50	0.13	0.63	0.10	0.10	0.062	0.12	0.40
**72**	CH	H	OMe	OMe	OMe	0.89	0.78	0.063	0.096	0.24	0.50	0.064	0.078	0.37
**101**	CH	H	OMe	H	OMe	0.96	0.77	0.69	0.64	0.78	0.88	0.65	0.84	0.59
**102**	CH	H	OMe	OMe	H	0.72	0.63	0.21	0.059	0.43	0.35	0.30	0.24	0.42
**103**	CH	OMe	H	OMe	H	0.091	0.24	0.030	0.12	0.65	0.40	0.034	0.032	0.26
**104**	CH	OMe	H	H	OMe	1.00	1.00	0.99	0.98	0.91	0.99	0.97	0.93	0.79
**105**	CH	H	H	OMe	H	0.0067	0.18	0.036	0.16	0.69	0.61	0.050	0.11	0.30
**106**	CH	H	OMe	H	H	0.69	0.25	0.089	0.24	0.085	0.90	0.27	0.62	0.37
**107**	CH	OMe	H	H	H	1.00	1.00	1.00	1.00	0.94	0.98	0.97	0.99	0.81
**108**	CH	H	OCH_2_O		H	0.98	1.00	1.00	0.99	0.87	0.98	0.96	0.98	0.69
**109**	CH	H	OCH_2_CH_2_O		H	0.37	0.90	0.44	0.43	0.40	0.79	0.19	0.49	0.47
**110**	N	H	OMe	OMe	OMe	0.81	0.43	0.065	0.38	0.75	0.80	0.47	0.47	0.45
**111**	N	H	OMe	H	OMe	0.013	0.062	0.063	0.018	0.37	0.41	0.026	0.020	0.25
**112**	N	H	H	OMe	H	0.0021	0.0032	0.19	0.0067	0.37	0.24	0.003	0.023	0.18

^a^ Abbreviations from columns left to right. CM: Cell morphology; CMI: Cell membrane integrity; ROS: Reactive oxygen species; GSH: Glutathione; NMI1: Nuclear membrane integrity 1; CC: Cell Cycle; NMI2: Nuclear membrane integrity 2; MMP: Mitochondrial membrane potential; CHI: Cell Health Index. ^b^ Traffic light colouring matching classifier values (Red–Green: high toxicity risk–low toxicity risk). ^c^ all *n* = 2.

## Data Availability

Not applicable.

## Supplementary Materials

The supporting information can be downloaded at: https://www.mdpi.com/article/10.3390/ijms24065088/s1.
