# Supplementary material for: Utilization of Supervised Machine Learning to Understand Kinase Inhibitor Toxophore Profiles
Source: Int J Mol Sci. 2023 Mar 7;24(6):5088. doi: 10.3390/ijms24065088 (PMC10049021; doi:10.3390/ijms24065088)
Supplement: Supplementary file 1 [file ijms-24-05088-s001.zip › ijms-2197815-supplementary.pdf]

Supporting Information

Utilization of Supervised Machine Learning to Understand Kinase Inhibitor Toxophore Profiles

Andrew A. Bieberich and Christopher R. M. Asquith *

*Email: christopher.asquith@uef.fi

Contents

1. Toxicity Profiling Platform - Representative Training Set

2. Toxicity Profiling Platform - Flow Cytometry Toxicity Profiling.

3. Toxicity Profiling Platform - Training Set Validation

4. Mass Spectrometry Method.

1. Toxicity Profiling Platform - Representative Training Set.

Table S1. Representative sample of compound training set outcome classes for Cell Health Screen.

Name	Cell Health Toxcity Profile[a,b]									
	CM	CMI	ROS	GSH	NMI1	CC	NMI2	MMP	CHI	OC
Tamoxifen	1.00	1.00	1.00	1.00	1.00	0.98	0.99	1.00	0.94	yes
Terfenadine	1.00	1.00	1.00	1.00	0.99	0.98	0.99	1.00	0.92	yes
Prochlorperazine	1.00	1.00	1.00	1.00	1.00	0.96	0.99	1.00	0.9	yes
Thioridazine	1.00	1.00	1.00	1.00	1.00	0.89	1.00	1.00	0.85	yes
Berberine	0.99	0.050	1.00	0.93	0.99	0.99	0.97	1.00	0.82	yes
Nefazodone	0.97	1.00	1.00	0.99	0.73	0.97	0.87	1.00	0.78	yes
Promethazine	1.00	0.98	0.98	0.96	0.84	0.46	0.94	0.97	0.71	yes
Rotenone	0.93	0.83	0.94	0.79	0.54	0.70	0.76	0.99	0.66	yes
Amiodarone	0.97	0.80	0.19	0.71	0.36	0.96	0.55	0.87	0.60	yes
Celecoxib	0.56	0.94	0.96	0.36	0.53	0.61	0.51	0.51	0.54	yes
Loratadine	0.36	0.70	0.85	0.64	0.33	0.84	0.52	0.65	0.42	no
Phenformin	0.74	0.27	0.77	0.17	0.82	0.28	0.080	0.37	0.41	no
Rosiglitazone	0.49	0.23	0.070	0.53	0.23	0.27	0.27	0.050	0.37	no
Nilutamide	0.040	0.060	0.090	0.040	0.21	0.68	0.52	0.42	0.34	no
Tranilast	0.020	0.13	0.12	0.030	0.73	0.10	0.30	0.17	0.32	no
Phentolamine	0.00	0.16	0.11	0.17	0.64	0.040	0.79	0.29	0.30	no
Propranolol	0.060	0.20	0.18	0.11	0.69	0.24	0.23	0.010	0.27	no
Sulfasalazine	0.010	0.060	0.090	0.030	0.14	0.10	0.63	0.31	0.25	no
Simvastatin	0.030	0.080	0.11	0.41	0.29	0.12	0.10	0.060	0.24	no
Buspirone	0.060	0.040	0.05	0.080	0.070	0.11	0.040	0.020	0.22	no
Diphenhydramine	0.010	0.030	0.040	0.030	0.20	0.030	0.11	0.030	0.20	no
Zileuton	0.020	0.010	0.070	0.020	0.17	0.11	0.080	0.060	0.19	no
Nevirapine	0.020	0.050	0.28	0.030	0.020	0.070	0.040	0.010	0.18	no
Fexofenadine	0.00	0.040	0.16	0.030	0.22	0.020	0.010	0.020	0.18	no
Pirfenidone	0.00	0.090	0.020	0.040	0.040	0.060	0.030	0.010	0.17	no
Ribavirin	0.010	0.070	0.23	0.060	0.16	0.040	0.030	0.000	0.16	no
Telbivudine	0.010	0.040	0.030	0.070	0.11	0.020	0.050	0.010	0.15	no
Clonidine	0.00	0.030	0.040	0.10	0.15	0.060	0.040	0.010	0.14	no
Oxaprozin	0.00	0.090	0.16	0.030	0.040	0.060	0.12	0.000	0.14	no
Ketoprofen	0.00	0.030	0.18	0.010	0.11	0.050	0.010	0.010	0.13	no

[a]Abbreviations from columns left to right. CM: Cell morphology; CMI: Cell membrane integrity; ROS: Reactive oxygen species; GSH: Glutathione; NMI1: Nuclear membrane integrity 1; CC: Cell Cycle; NMI2: Nuclear membrane integrity 2; MMP: Mitochondrial membrane potential; CHI: Cell Health Index. [b]all n=2

The AsedaSciences SYSTEMETRIC Cell Health Screen uses a supervised machine learning classifier to estimate human safety risk for small molecule compounds. The classifier was trained on a set of 300 known compounds, including on-market and withdrawn pharmaceuticals, research compounds, and a few agricultural/industrial compounds. All training compounds were curated using research literature and market/clinical histories, where applicable. Curation enabled the assignment of each compound to an outcome class (OC, far right column). The "yes" class includes all compounds expected, based on external information, to cause elevated cell stress phenotypes as a result of documented cytotoxicity mechanisms and/or poor human safety profiles. The "no" class includes compounds expected to cause little to no cell stress. All training compounds were then processed through the Cell Health Screen to produce flow cytometry-based phenotypes, as described in Methods. Class and phenotypic data were then used to optimize a multidimensional logistic regression model

describing the dependence of class membership upon phenotype strength. For any unknown test compound, the trained classifier outputs the probability that the test compound phenotype belongs in the "yes" class, essentially quantifying the test compound's phenotypic similarity to the known high-safety risk compounds in the training set. This probability is the Cell Health Index. The 30 compounds listed above are a representative sample from the training set for purposes of illustrating the types of compounds included and their phenotypic range. Column header key: CM=cell morphology, CMI=cell membrane integrity, ROS=reactive oxygen species (with some specificity for mitochondrial superoxide), GSH=glutathione, NMI1=nuclear membrane integrity 1, CC=cell cycle, NMI2= nuclear membrane integrity 2, MMP=mitochondrial membrane polarization, and CHI=Cell Health Index. For the eight endpoint scores other than CHI, the score is calculated by applying the classifier model *only* to the flow cytometry parameter(s) informing each endpoint.

2. Toxicity Profiling Platform - Flow Cytometry Toxicity Profiling.

- Previously Reported in Reference 24 and 25 of the main manuscript.

AsedaSciences SYSTEMETRIC® Cell Health Screen

1. Physical execution summary

In a 384-well platform, HL60 cells were exposed to a 10-step, 3x dilution series of each test compound (5 nM-100 μM) for 4 hours at 37°C with 5% CO_2. Each dilution series was screened in duplicate, occupying a total of 20 wells, allowing 16 test compounds to be assayed per plate. Each row contained one positive and one negative control well, for a total of 16 replicate positive/negative control pairs on each assay plate. Compound formatting, cell deposition, and dye application were performed robotically, so that final assay conditions comprised 100,000 cells in a 40 μL volume. After the 4-hour compound exposure, cells were immediately stained with a panel of fluorescent dyes that reported physiological signatures of both mitochondrial dysfunction and gross cell stress. Fluorescence and forward/side-scatter data were collected using automated FC with no gating. FC data are processed by an automated algorithm for producing quality control measures and ML classification of compound phenotypes.

2. HL60 cell culture production

HL60 cells were produced as suspension cultures in glass 850cm^2 roller bottles with vented caps at 1 RPM, 5% CO_2, and 37°C. The culture medium was RPMI 1640 without glucose, supplemented with 10mM galactose and 10% dialyzed heat-inactivated FBS (Atlanta Biologicals). Culture density was maintained at or below 1x10^6 cells/mL. The standard protocol for the Cell Health Screen is that a new production lineage of HL60 cells is started each month, and a crossover screen is performed in which the old and new production lineages are compared by using a set of 16 reference compounds to produce a known set of stress phenotypes (see supporting information). In this way, variation of screen performance is minimized by producing all screening cell populations within a narrow range of passage numbers, each checked for consistency of phenotypic performance with reference compounds. This process was performed prior to using cells to produce data for this study.

3. Test compound format, cell exposure, and staining

All compound formatting, cell exposure, and staining with reporter dyes were performed in this study according to a standard protocol for the Cell Health Screen, which is described as follows. Compounds were formatted in groups of 16, with DMSO blanks loaded in unused screen positions for any smaller compound groups required to finish the complete study set. Each set of 16 test compounds was formatted in two replicate 384-well plates (Eppendorf Protein LoBind®, catalog number 951040589) for assays with two subsets of fluorescent dyes (Spectral overlap and DMSO limitation prevent simultaneous use of the complete dye panel in a single plate.). Compounds in these replicate plates

were identical except for the positive controls, which were chosen to produce an optimal response within each subset of fluorescent reporter dyes. Test compound dilution series and controls were formatted on a Biomek® 4000. Each compound was formatted as a 10-step, 3x dilution series, in duplicate, on each of the two plates. Negative control wells contained the diluent used for both the test compound dilution series and positive controls. The diluent was RPMI 1640 (supplemented as above) with a final working concentration of DMSO normalized to 1% in all wells. The positive and negative controls were distributed to plate wells from a single initial reservoir of each control mixture. The final assay concentration range for test compounds was 5 nM-100 µM. Prior to cell deposition, assay plates containing formatted compounds were sealed and stored at room temperature, protected from light, for 2 hours to allow binding equilibrium between serum components and test compounds. A Biomek NX^P (Beckman Coulter) was used to deposit cells in all wells, at a density of 2.5×10^6 cells/mL, in a final assay volume of 40 µL per well (approximately 100,000 cells per well). After cell deposition, each assay plate was sealed with breathable plate sealer, shaken at 2,200 RPM for 10 seconds (Illumina® High-speed microplate shaker), and incubated for 4 hours at 37°C with 5% CO_2.

3a. First fluorescent dye mix staining conditions

The following protocol was applied to the first plate in each assay plate pair, inclusive of all compounds in this study. The dye mix buffer was 1x PBS with 4% FBS, filter sterilized. The dye set consisted of Calcein AM, SYTOX™ Red, MitoSOX™ Red, and Monobromobimane (Life Technologies catalog numbers C1430, S34859, M36008, and M20381, respectively). Dye concentrations were previously optimized, during the screen prototyping phase, to produce a maximum dynamic range between positive and negative control wells. Prior to the deposition of the dye mix, the assay plate was removed from its 4-hour incubation, and cells were gently pelleted at 300xg for 2 minutes. A Biomek NX^P (Beckman Coulter) was then used to aspirate 20 µL of each well volume, after which 20µL of the dye mix was deposited in all wells. After dye deposition, the plate was re-sealed with its breathable plate sealer, shaken 2x at 2,200 RPM for 5 seconds each time (1-second interval), and incubated for 10 minutes at 37°C with 5% CO_2. The plate was then rapidly cooled to room temperature for 1 minute in a shallow water bath, after which the acquisition of flow cytometry data was started immediately.

3b. Second fluorescent dye mix staining conditions

The following protocol was applied to the second plate in each assay plate pair, inclusive of all compounds in this study. The dye mix buffer was 1x PBS with 4% FBS, filter sterilized. The dye set consisted of JC-9, propidium iodide, and Vybrant® DyeCycle™ Violet (Life Technologies catalog numbers D22421, P3566, and V35003, respectively). Dye concentrations were previously optimized, during the screen prototyping phase, to produce a maximum dynamic range between positive and negative control wells. Cell pelleting and dye deposition were performed as above, in 3a. After dye deposition, the plate was re-sealed with its breathable plate sealer, shaken 2x at 2,200 RPM for 5 seconds each time (1-second interval), and incubated for 30 minutes at 37°C with 5% CO_2. The plate was then allowed to sit at room temperature for 15 minutes, protected from light. Acquisition of flow cytometry data was started immediately after this 15-minute period.

4. Acquisition of flow cytometry data

FC data were acquired with a CyAn™ ADP flow cytometer (Beckman Coulter) with automated sampling performed by a HyperCyt® autosampler (Intellicyt). Autosampler settings were optimized to aspirate ≥10,000 cells per well. As described in Section 3 above, the complete set of fluorescent dyes was applied as two non-overlapping mixtures on replicate assay plates. Therefore, two separate FC acquisition protocols with different sets of detection channels were used. Note that all channels were acquired with no gating. Triggering was on Forward Scatter with Threshold = 5%. Acquisition channel settings in Summit (version 4.3) for these two protocols are reported in Bieberich *et al.* [24].

5. Data processing and analysis

All well-specific FC data and matching plate map files were transferred to an EC2 server instance on Amazon Web Services (AWS). An automated algorithm converts the raw data to risk scores for each compound in two stages:

5a. Feature reduction

For each test of each compound, all 12 ungated FC detection parameters were converted to a feature vector as follows. For each of the ten concentration steps in a test compound dilution series, quadratic form (QF) distance was calculated between the empirical distribution of an FC detection parameter and that parameter in the negative control [24]. This effectively quantitates the amount of change in test cells relative to the negative control cells that each concentration of the test compound caused in one of the 12 FC detection channels. For each FC detection parameter, the amount of change between each test well and the negative control was thus converted to a dose-response curve of QF distance values. The same process was executed for all 12 FC detection parameters, after which each of the 12 QF distance value curves was further reduced to two values: the point of the maximum rate of change and the range within which change occurs.[26] These two feature values for each FC parameter were then assembled into a vector representing all 12 FC parameters. This vector serves as the quantitative digital phenotype for the test compound to be used in subsequent ML classification [24].

5b. Machine learning classification

Risk scores were produced for test compounds with an ML classifier employing supervised learning, with a multidimensional logistic model regularized by an elastic net. The classifier was trained on a set of 300 known compounds drawn from on-market pharmaceuticals, withdrawn drugs, research compounds, and a few industrial/agricultural compounds (representative set shown in **Table S1**). First, all training set compounds were assigned to one of two binary outcome classes: the "yes" (expectation of high cell stress) or "no" class. This assignment was based on manually curated external information from the scientific literature, clinical trial results, and commercial histories (where applicable). Each training compound was also screened to produce an empirical phenotypic feature vector, as described above. In this way, each of the two outcome classes in the training set was populated with an empirical distribution of cell-stress phenotypes from the FC screen. With these two data types attached to each training compound, historical outcome and empirical cellular phenotype, the goal of classifier training was to quantify the dependence of class membership on phenotype. This is a classic problem for an optimized logistic regression model. The classifier was trained by repeated cross-validation. Using the two training outcome classes, the logistic model optimization process sought the most parsimonious model allowing for maximum separation of the two populations of phenotypes. The optimally fit model then became the classification tool, allowing calculation of the probability that a feature vector from any compound could be assigned to the "yes" (high cell stress) class. Subsequently, for any test compound, the final risk score, or Cell Health Index (CHI), was the probability (maximum likelihood) with which the test compound's phenotypic feature vector could be assigned to the "yes" class defined by the training set. In addition, a series of lower-dimensional classifiers were trained on the same training set, calculating the probability of a "yes" class assignment if only data for specific endpoints were considered. For example, the two FC detection parameters, forward-scatter and side-scatter, from the 488nm laser, were input to the classifier to produce the score called "cell morphology" (CM). These endpoint classifications produced a "biological fingerprint" of scores that can be interpreted as indicating the relative contributions of each endpoint to the final multiparameter CHI score. However, note that the predictivity of the individual endpoints is not assumed to be equal among themselves or to the CHI.

3. Toxicity Profiling Platform - Training Set Validation

Table S2. Representative AsedaSciences SYSTEMETRIC® Cell Health Screen Performance Validation.

Calibrated to Human Clinical Outcome

n = 369	Reference: No	Reference: Yes
Prediction: No	261	12
Prediction: Yes	6	90

Acute Cell Stress	Performance
Accuracy	0.9512
95% CI	0.924, 0.9708
Sensitivity	88%
Specificity	98%
PPV	94%
NPV	96%

All Toxicity	Performance
Accuracy	0.826
95% CI	0.786, 0.862
Sensitivity	63%
Specificity	94%
PPV	86%
NPV	81%

Abbreviations: PPV = Positive Predictive Value and NPV = Negative Predictive Value.

4. Mass Spectrometry Method.

Samples were analyzed with a ThermoFisher Q Exactive HF-X (ThermoFisher, Bremen, Germany) mass spectrometer coupled with a Waters Acquity H-class liquid chromatograph system. Samples were introduced via a heated electrospray source (HESI) at a flow rate of 0.6 mL/min. Electrospray source conditions were set as: spray voltage 3.0 kV, sheath gas (nitrogen) 60 arb, auxiliary gas (nitrogen) 20 arb, sweep gas (nitrogen) 0 arb, nebulizer temperature 375 degrees C, capillary temperature 380 degrees C, RF funnel 45 V. The mass range was set to 150-2000 m/z. All measurements were recorded at a resolution setting of 120,000.

Separations were conducted on a Waters Acquity UPLC BEH C18 column (2.1 x 50 mM, 1.7 µm particle size). LC conditions were set at 100 % water with 0.1 % formic acid (A), ramped linearly over 9.8 mins to 95 % acetonitrile with 0.1 % formic acid (B) and held until 10.2 mins. At 10.21 mins, the gradient was switched back to 100% A and allowed to re-equilibrate until 11.25 mins. The injection volume for all samples was 3 uL.

Xcalibur (ThermoFisher, Breman, Germany) was used to analyze the data. Solutions were analyzed at 0.1 mg/mL or less based on responsiveness to the ESI mechanism. Molecular formula assignments were determined with Molecular Formula Calculator (v 1.2.3). All observed species were singly charged, as verified by unit *m*/*z* separation between mass spectral peaks corresponding to the ^{12}C and $^{13}C^{12}C_{c-1}$ isotope for each elemental composition.
